# Berberine–cinnamic acid co‐crystal effect in ameliorating hyperlipidemia might be regulated through the PI3K/AKT/mTOR/SREBP‐1 signaling pathway

**DOI:** 10.1002/2211-5463.70115

**Published:** 2025-09-10

**Authors:** Wenheng Gao, Yunlong Li, Lihua Chen, Wenshuo Yang, Yong He, Ye Yang, Dengke Yin, Song Tan

**Affiliations:** ^1^ School of Pharmacy Anhui University of Chinese Medicine Hefei China; ^2^ Anhui Provincial Key Laboratory of Traditional Chinese Medicine Formula Huaibei China; ^3^ Anhui Provincial Key Laboratory of Chinese Medicinal Formula Hefei China

**Keywords:** berberine–cinnamic acid co‐crystal, hyperlipidemia, lipid, PI3K/AKT/mTOR, SREBP‐1

## Abstract

Hyperlipidemia is a common chronic disease characterized by elevated levels of lipids in the blood. There is some evidence that suggests that berberine (BBR) might be beneficial for the treatment of hyperlipidemia. However, its low intestinal bioavailability limits its potential therapeutic action. In the present study, we explored the effect and the underlying mechanism of berberine–cinnamic acid co‐crystal (BBR‐CA), which is self‐assembled from CA and BBR and displays a high intestinal bioavailability. In mice, BBR‐CA showed the ability to decrease body weight gain and hepatic lipid accumulation in animals fed a high‐fat diet. To further characterize the molecular basis of this effect, we established a hyperlipidemia cell model by treating human hepatocellular carcinoma cells (HepG2) with free fatty acids. Similarly to our *in vivo* experiments, lipid accumulation in free fatty acids‐induced HepG2 cells was also reduced by BBR‐CA. We hypothesized that BBR‐CA might act through the regulation of sterol regulatory element‐binding proteins‐1 (SREBP‐1), a key factor regulating lipid synthesis, and, indeed, SREBP‐1 protein expression was inhibited by BBR‐CA treatment, resulting in the decreased expression of its downstream proteins stearoyl‐CoA desaturase 1 and acetyl‐CoA carboxylase. Furthermore, the phosphorylation of phosphatidylinositol 3‐kinase (PI3K), AKT and mammalian target of rapamycin (mTOR) was inhibited by BBR‐CA, contributing to decreased active SREBP‐1 in the nucleus, and was reversed and enhanced by the PI3K agonist recilisib and inhibitor LY294002, respectively. Taken together, our results suggest that BBR‐CA could function by modulating the PI3K/AKT/mTOR signaling pathway, resulting in decreased nuclear expression of SREBP‐1, as well as reduced expression of stearoyl‐CoA desaturase 1 and acetyl‐CoA carboxylase, thus alleviating hyperlipidemia. Further experimental validation is required to confirm these results.

## Graphical abstract

Berberine–cinnamic acid co‐crystal (BBR‐CA) inhibits the phosphorylation of the phosphatidylinositol 3‐kinase (PI3K)/AKT/mammalian target of rapamycin (mTOR) pathway, suppressing the transfer of pre‐sterol regulatory element‐binding proteins‐1 (SREBP‐1) from the endoplasmic reticulum to the nucleus. This results in a decrease in the expression level of nSREBP‐1 within the nucleus, and further affects the functions of downstream lipases such as stearoyl‐CoA desaturase 1 (SCD1) and acetyl‐CoA carboxylase (ACC), thereby influencing lipid synthesis.

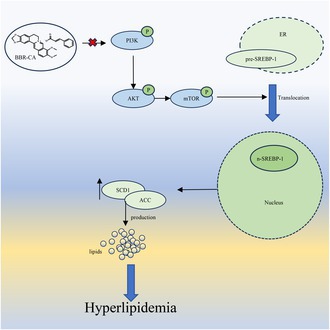

**Abbreviations**
ACC: acetyl‐CoA carboxylase
ALT: alanine aminotransferase
ANOVA: analysis of variance
AST: aminotransferase
AT: atorvastatin
BBR: berberine
BBR‐CA: berberine–cinnamic acid co‐crystal
CA: cinnamic acid
CCK‐8: Cell Counting Kit‐8
CTL: control
FFA: free fatty acids
H&E: hematoxylin and eosin
HDL‐C: high‐density lipoprotein cholesterol
HFD: high‐fat diet
LDL‐C: low‐density lipoprotein cholesterol
mTOR: mammalian target of rapamycin
NFD: normal food diet
p: phosphorylated
PI3K: phosphatidylinositol 3‐kinase
SCD1: stearoyl‐CoA desaturase 1
SREBP‐1: sterol regulatory element‐binding protein‐1
TC: total cholesterol
TG: triglycerides

Hyperlipidemia, a common chronic disease, mainly characterized by elevated levels of total cholesterol (TC) and triglycerides (TG) in the blood [1]. The main clinical consequences of hyperlipidemia are an increased susceptibility to atherosclerosis and related cardiovascular diseases [2, 3]. The causes of hyperlipidemia are diverse, such as unhealthy eating habits, psychological factors, diabetes, pathological conditions and heredity [4, 5, 6]. Treatment strategies include lifestyle modifications, such as dietary adjustments and consistent physical exertion, as well as pharmacological interventions such as statins, fibrates and niacin [1, 7, 8]. Despite these measures, hyperlipidemia remains a formidable health challenge, highlighting the urgency of continuously exploring innovative treatment methods and prevention strategies.

Chinese herbal medicines and their active ingredients play an irreplaceable role in regulating blood lipids [9]. Berberine (BBR) is a quaternary ammonium salt alkaloid isolated from *Rhizoma coptidis* (Huanglian), and it is commonly used as a broad‐spectrum antibacterial drug. Meanwhile, both preclinical and clinical studies have demonstrated that BBR is beneficial for the treatment of hyperlipidemia [10, 11]. However, BBR has a low intestinal bioavailability [12], requiring high doses and/or long‐term treatment, which usually causes unwanted side effects (such as constipation, nausea and abdominal distension), limiting its therapeutic action on hyperlipidemia [13]. From the Huanglian‐*Cinnamomum cassia* (Rougui) herb pairs, which were first recorded in ‘Han's Medical Circular’ and named as the Jiaotai Pill to treat diabetes, a nanoparticle composed of BBR and cinnamic acid (CA) was directly self‐assembled. In previous research, a BBR‐CA co‐crystal and its series of derivatives were synthesized using BBR and CA, and it increased the solubility and bioavailability of BBR, demonstrating a significant therapeutic effect in mouse models of colitis and diabetes [14, 15, 16]. However, its effect of BBR‐CA on the treatment of hyperlipidemia and the underlying mechanism remains unknown.

Sterol regulatory element‐binding protein‐1 (SREBP‐1) is the key nuclear transcription factors that enhances the transcription of genes required for lipid synthesis, play an important role in hyperlipidemia [17, 18]. SREBP‐1 regulates fatty acid synthesis and cholesterol synthesis by modulating the expression of genes related to lipid synthesis. For example, SREBP‐1 can regulate lipid synthesis by influencing the expression of acetyl‐CoA carboxylase (ACC) and stearoyl‐CoA desaturase 1 (SCD1) [19]. The phosphorylation of phosphatidylinositol 3‐kinase (PI3K)/AKT/mammalian target of rapamycin (mTOR)/SREBP‐1 signaling pathway is a key regulator of lipid metabolism in hepatocytes [20]. When the body is stimulated by a high‐fat diet (HFD), these stimuli can lead to the activation of AKT by PI3K. AKT enhances the expression of SREBP‐1 and promotes nucleic acid accumulation by activating mTOR, thus accelerating lipid production. In the state of hyperlipidemia, the activity of SREBP‐1 is often enhanced, resulting in increased lipid synthesis and further aggravating dyslipidemia. Therefore, in recent years, many studies have focused on the PI3K/AKT/mTOR/SREBP‐1 signaling pathway and attempted to identify inhibitors of the PI3K/AKT/mTOR/SREBP‐1 signaling pathway to control the occurrence and development of hyperlipidemia [21, 22, 23, 24].

In the present study, hyperlipidemia mouse and lipid‐riched cell models were established. Through *in vivo* and *in vitro* experiments, it was confirmed that BBR‐CA has a significant therapeutic effect on hyperlipidemia. Moreover, it can affect the expression of SREBP‐1 in the nucleus through the PI3K/AKT/mTOR pathway, and consequently influence the expression of downstream lipogenesis genes such as SCD1 and ACC, thus achieving the purpose of treating hyperlipidemia.

## Materials and methods

### Antibodies and reagents

Dulbecco's modified Eagle medium, BSA, penicillin–streptomycin, 0.25% trypsin solution, Cell Counting Kit‐8 (CCK‐8), a color‐enhanced protein molecular marker (EC0019) and 10% SDS/PAGE preparation kit (EC0023), poly(vinylidene difluoride) membranes (ED0005), a radioimmunoprecipitation assay, a bicinchoninic acid protein assay kit, and Sparkjade ECL super, recilisib (SJ‐MX5040A), LY294002 (SJ‐MX0083) and substrate were purchased from Shandong Sparkjade Biotechnology Co., Ltd (Shandong, China). Fetal bovine serum was obtained from Biochannel (Nanjing, China). The primary antibodies used for western blotting and immunohistochemistry are listed in Table S1. The secondary antibody, goat anti‐rabbit IgG (Alexa Fluor 488), was purchased from Shanghai Abway Biotechnology Co., Ltd (Shanghai, China). The Nuclear Protein Extraction Kit was purchased from Shanghai Biyuntian Biotechnology Co., Ltd (Shanghai, China). The synthesis of BBR‐CA and the detection of related indicators are as described in the references [14‐16]. In brief, BBR and CA (1 : 1 molar ratio, BBR 500 mg) were dissolved in 30 mL of MeOH. The solvent was removed with a rotary evaporator under vacuum condition of 45 °C. Yellow solids were obtained and dried in a vacuum oven at 40 °C for 2 h. The vapor diffusion method (MeOH:AcOEt) was used to scale up the co‐crystal preparation [14, 15, 16]. Single‐crystal X‐ray diffraction data were collected at 193 K on a SMART APEX II diffractometer (Bruker, Billerica, MA, USA) (Cu Kα radiation). The structure was solved with shelxt (http://shelx.uni‐goettingen.de/) and refined anisotropically for non‐H atoms using olex2 (https://www.olexsys.org/olex2/). Hydrogen atoms were located from difference Fourier maps and refined using a riding model. Disordered solvent was treated via platon squeeze (http://www.platonsoft.nl/platon/pl000000.html) (space group P2₁/*c*, Z′ = 1), removing 149 electrons per unit cell. Final refinement converged at *R*₁ = 0.05 (293 K). The crystal structure was visualized with diamond (https://www.crystalimpact.de/diamond).

### Animals and treatment

All animal experiments conducted in this study were approved by the Animal Ethics Committee of Anhui University of Chinese Medicine (Ethics Number: AHUCM‐mouse‐2 023 156). Male C57BL/6J mice, aged 5 weeks, were obtained from Hangzhou Ziyuan Laboratory Animal Technology Co., Ltd (Hangzhou, China). The mice were subjected to a 12 : 12 h light/dark photocycle at 22 ± 2 °C and 55% relative humidity, and were provided with water and food *ad libitum*. Before the start of the experiment, the mice were allowed to acclimate for 1 week. Then, the mice were fed a normal food diet (NFD) and a HFD (MD12032; Jiangsu Medison Biopharmaceutical Co., Ltd, Jiangsu, China) for 8 weeks, with 8 mice in each group (*n* = 8). During this period, atorvastatin (AT) (10 mg·kg^−1^), BBR (70 mg·kg^−1^) and BBR‐CA (low dose 50 mg·kg^−1^; medium dose 100 mg·kg^−1^; high dose 150 mg·kg^−1^) were administered for intervention treatment by gavage. The NFD group and the HFD group were given the same volume of distilled water. Animals were humanely euthanized‐under deep surgical isoflurane anesthesia (5%). Animals underwent cardiac puncture to collect terminal blood samples. The mice were dissected and samples were collected for the next experiment.

### Hematoxylin and eosin (H&E) staining

The fresh liver, heart, spleen, lungs and kidneys of mice were preserved in 4% paraformaldehyde solution, and their histological changes were evaluated using the H&E staining method.

### Biochemical analysis

Kits were e,ployed in accordance with the manufacturer's instructions (Shanghai Jiang Lai Bio‐Technology Co., Ltd, Shanghai, China) to detect the levels of TC, TG, low‐density lipoprotein cholesterol (LDL‐C) and high‐density lipoprotein cholesterol (HDL‐C) in the blood, as well as the levels of aspartate aminotransferase (AST) and alanine aminotransferase (ALT) in the liver.

### Western blotting

Liver tissues and cells were lysed in radioimmunoprecipitation assay buffer supplemented with a protease inhibitor cocktail and a phosphatase inhibitor cocktail (Shandong Sparkjade Biotechnology Co., Ltd) and extract the nuclear proteins using the Nuclear Protein Extraction Kit (Shanghai Biyuntian Biotechnology Co., Ltd). Equal amounts of the lysates were separated by SDS/PAGE electrophoresis and transferred onto poly(vinylidene difluoride) membranes.

### Immunofluorescence

To investigate the expression of SREBP‐1 protein within the cell nucleus, an immunofluorescence assay was carried out. First, the cells cultured on the treated coverslips were harvested and fixed. Subsequently, permeabilization was performed using 0.2% Triton X‐100 (Saiguo Biotechnology Co., Ltd, Guangzhou, China) to facilitate antibody penetration. Following this, blocking was achieved with 5% BSA to minimize non‐specific binding. After the completion of the blocking process, the primary antibody specific to SREBP‐1 was added, and the samples were incubated overnight at 4 °C. The next day, the fluorescent secondary antibody conjugated with Alexa Fluor 488 was introduced, and the samples were incubated at room temperature under light‐protected conditions. Subsequently, nuclear staining was conducted using DAPI (i.e. 4′,6‐diamidino‐2‐phenylindole) staining solution under dark conditions. Finally, the samples were mounted with an anti‐fluorescence quenching mounting medium, and images were visualized and captured under a fluorescence inverted microscope.

### Quantitative real‐time PCR

Total RNA was isolated from the treated cells and liver using an RNA extraction kit (OSR‐M610; TIANGEN, Beijing, China). Next, 2 μg of RNA was used to synthesize cDNA using a cDNA synthesis kit (TaKaRa, Otsu, Japan). The primer sequences used in these experiments are listed in Table S2. The specificity of primers was confirmed by analyzing the characteristics of the PCR curve and the change in fluorescence intensity before the experiment. The fold change of genes was calculated using the ∆∆C_t_ method [25].

### Cell culture and treatments

HepG2 cells (Cell Resource Center of Shanghai Institutes for Biological Sciences, Chinese Academy of Sciences, Shanghai, China) were cultured in Dulbecco's modified Eagle medium supplemented with 10% (v/v) fetal bovine serum and 1% (v/v) penicillin–streptomycin. The cells were incubated at 37 °C in a 5% CO_2_ incubator.

HepG2 cells (1.1 × 10^4^ cells per well) were seeded into 96‐well plates and incubated at 37 °C for 24 h. A hyperlipidemia cell model was established by treating human hepatocellular carcinoma cells (HepG2) with free fatty acids (FFA; sodium oleate and sodium palmitate at a ratio of 2:1; purchased from Shanghai Aladdin Biochemical Technology Co., Ltd, Shanghai, China) at a final concentration of 1 mm for 24 h. Meanwhile, the control group was incubated in normal medium. The cells were treated with drugs for 24 h before the model establishment. Subsequently, the cells were stained with Oil Red O (LEAGENE, Beijing, China) and the area of lipid droplets was observed under an inverted microscope. Then, the cells were decolorized with isopropanol, and absorbance was measured at 510 nm (for this, 100 μL of isopropanol was added, followed by shaking evenly on a shaker for 20 min, and absorbance was measured at 510 nm, which represents the lipid content). Under the same conditions, HepG2 cells (2.5 × 10^5^ cells per well) were seeded into six‐well plates and treated. After that, the content of TG was measured and western blotting experiments were carried out by extracting proteins.

The CCK‐8 kit was used to detect the effects of drugs and FFA on cell viability respectively, aiming to determine the concentrations of FFA and drugs in each group. We divided the cells into nine groups: the control group (CTL), the model group (FFA), the AT group (20 μm), the CA group (20 μm), the BBR group (20 μm), the CA + BBR group (20 μm), the low‐dose BBR‐CA co‐crystal group (L‐BBR‐CA) (10 μm), the medium‐dose BBR‐CA co‐crystal group (M‐BBR‐CA) (20 μm) and the high‐dose BBR‐CA co‐crystal group (H‐BBR‐CA) (30 μm). In addition, in the subsequent experiments, we also used the activator (recilisib, 10 μm) and inhibitor (LY294002, 10 μm) of PI3K to study the impact of BBR‐CA (10 μm) on the PI3K pathway.

### Statistical analysis

The data are presented as the mean ± SD and were analyzed by one‐way analysis of variance (ANOVA) Duncan's multiple range test for comparison experiments. *P* < 0.05 was considered statistically significant.

## Results

### The inhibitory function of BBR‐CA on hyperlipidemia *in vivo*

**Fig. 1:**
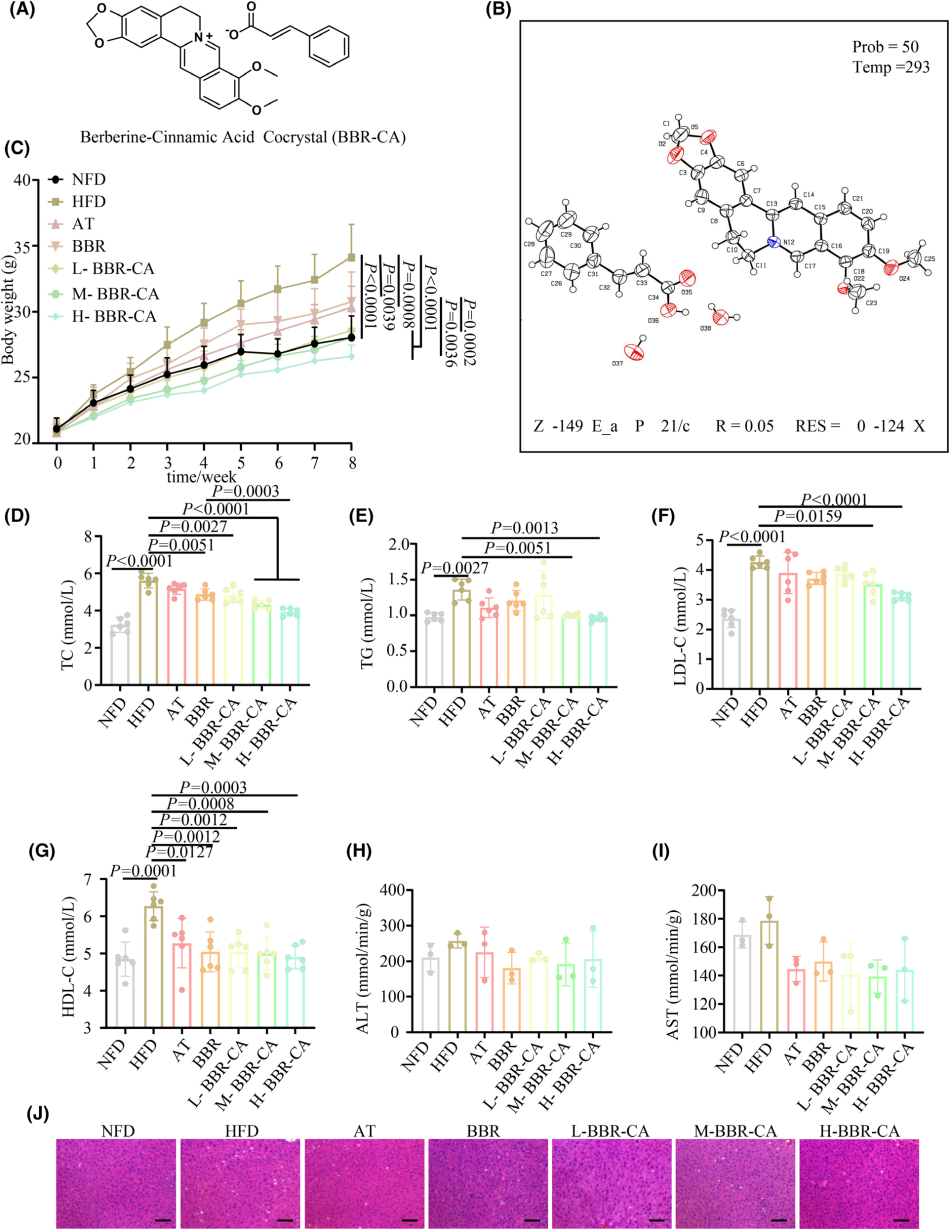
Characterization of BBR‐CA and its effect on hyperlipidemia mice. (A) The chemical structure of BBR‐CA. (B) Molecular structure determined by single‐crystal X‐ray diffraction analysis of BBR‐CA. Carbon atom positions (C1–C37) are labeled. The crystal was measured at 293 K and belongs to the monoclinic crystal system, space group P2₁/*c*. The structure refinement shows high precision with a reliability factor of *R* = 0.05. (C) Changes in body weight of mice within 8 weeks after drug treatment (*n* = 8, where *n* represents the number of animals). (D–G) The concentration of TC, TG, LDL‐C and HDL‐C in serum of mice (*n* = 6, where n represents the number of mouse serum samples). (H, I) The concentration of ALT and AST in the liver of mice (*n* = 3, where *n* represents the number of mouse liver samples). (J) H&E staining of the liver in mice; scale bar = 50 μm. The data are expressed as mean ± SD and analyzed by one‐way ANOVA with Duncan's multiple range test for comparative experiments. *P* < 0.05 was considered statistically significant.

The structural formula of BBR‐CA is shown in Fig. 1A and the X‐ray diffraction pattern of BBR‐CA is shown in Fig. 1B. The BBR‐CA co‐crystal was verified by X‐ ray powder diffraction technology, and the characteristic peaks of the obtained X‐ray diffraction spectrum appeared at 5.599, 8.121, 12.779, 14.532, 15.147, 16.286, 17.138, 17.471, 18.312, 19.733, etc. 22.733, 23.705, 24.221, 25.110, 25.362, 25.591, 25.948, 26.478, 27.355, 27.863, 28.184, 28.725, 29.840 and 30.225.

Following an 8‐week treatment period, a significant increase in body weight was observed in the mice of the HFD group compared to those in the NFD group. By contrast, the body weights of mice in each drug‐administered group were effectively controlled. As a positive control drug, AT significantly reduced the body weight of mice and improved lipid metabolism disorders. From the results, the overall effects of the BBR group and the BBR‐CA group with respect to improving mouse body weight and blood lipids were superior to those of AT (Fig. 1C–G). Notably, the H‐BBR‐CA group exhibited the most pronounced effect in body weight control, whereas the L‐BBR‐CA group demonstrated a comparable outcome to the BBR group (Fig. 1C). As previously reported, long‐term consumption of a HFD is frequently associated with abnormal lipid metabolism [1]. In the present study, HFD feeding led to disruptions in the levels of serum TC, TG, LDL‐C and HDL‐C compared to the NFD group (Fig. 1D–G). Upon supplementation with BBR‐CA, a significant reduction in the levels of TC, TG, LDL‐C and HDL‐C was noted compared to the HFD group. Remarkably, the efficacy of BBR‐CA in reducing TC and TG was substantially superior to that of BBR. Additionally, the levels of ALT and AST in the livers of mice were evaluated, and no significant differences were detected between each drug‐administered group and the HFD group (Fig. 1H,I). H&E staining revealed that the hepatic lobule structures in the NFD group were arranged in an orderly and compact fashion. By contrast, conspicuous lipid droplet accumulation was evident in the HFD group. However, following drug treatment, these pathological alterations were significantly ameliorated (Fig. 1J). With respect to the heart, spleen, lungs and kidneys, no significant differences were discernible among the groups, suggesting that BBR‐CA does not regulate body weight through the induction of overt toxicity in mice (Fig. S1). In summary, 8 weeks of HFD intervention precipitated the development of hyperlipidemia, yet supplementation with BBR‐CA effectively mitigated the dyslipidemia and hepatic lipid accumulation induced by HFD.

### BBR‐CA reduces the expression of lipid synthesis‐related proteins and genes in the liver

**Fig. 2:**
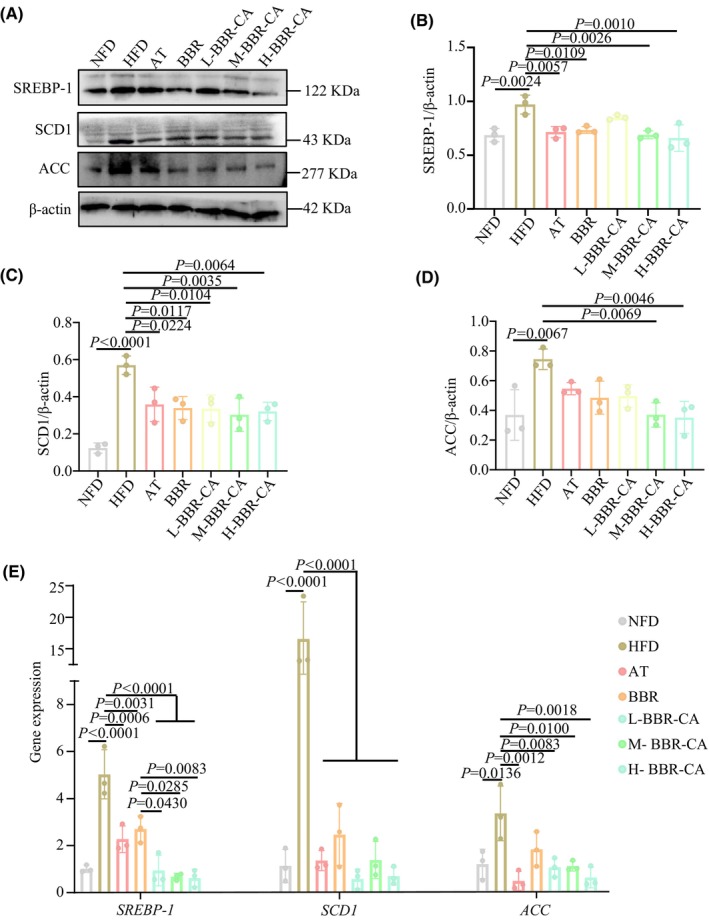
The effects of BBR‐CA on the expression of hepatic lipid synthesis‐related proteins and genes in hyperlipidemia mice. (A) Western blotting detects the protein expression of SREBP‐1, SCD1 and ACC in liver of mice. (B–D) Quantification of the SREBP‐1, SCD1 and ACC bands from (A) (*n* = 3, where *n* represents the number of mouse liver samples). (E) Changes in mRNA expression of *SREBP‐1*, *SCD1* and *ACC*, in liver of mice (*n* = 3, where *n* represents the number of mouse liver samples). The data are expressed as mean ± SD and analyzed by one‐way ANOVA with Duncan's multiple range test for comparative experiments. *P* < 0.05 was considered statistically significant.

Compared with the NFD group, the protein expression levels of SREBP‐1, SCD1 and ACC in the HFD group were significantly increased. After administration of BBR‐CA, the protein levels in the livers of mice were significantly decreased (Fig. 2A–D). Meanwhile, the gene expression levels of *SREBP‐1*, *SCD1* AND *ACC* in the HFD group were also significantly increased (Fig. 2E). After administration of BBR‐CA, the gene levels in the liver were all significantly downregulated. Among them, the expression level of SREBP‐1 in the M‐BBR‐CA group was significantly lower than that in the BBR group (Fig. 2E). The above results indicate that BBR‐CA can reduce the expression of proteins and genes involved in hepatic lipid synthesis.

### The inhibitory function of BBR‐CA on intracellular lipid accumulation in HepG2 cells

**Fig. 3:**
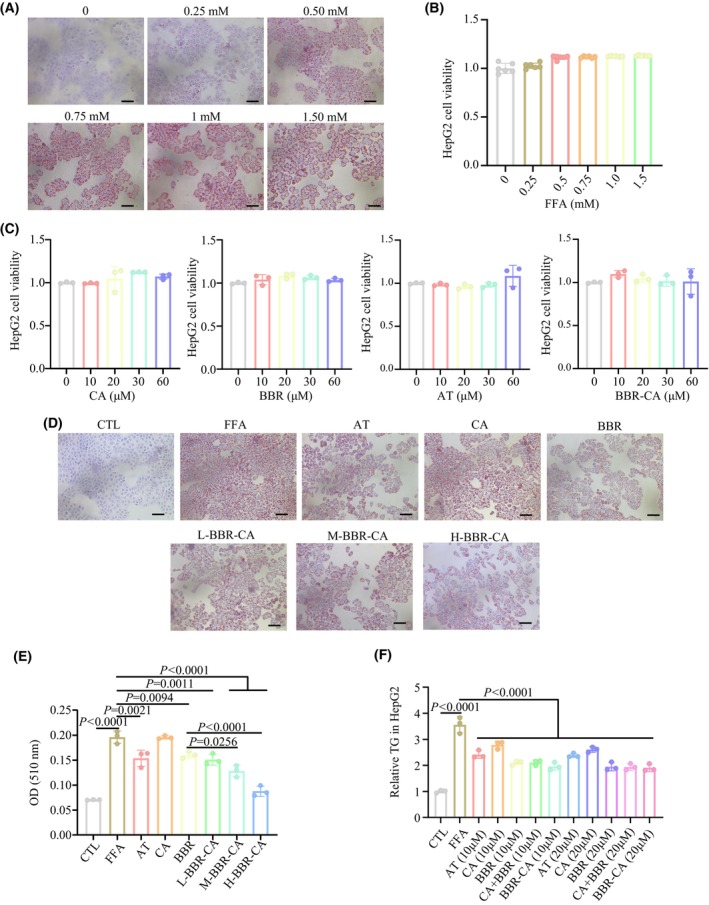
Establishment of FFA‐induced HepG2 cell model and the effect of BBR‐CA on lipid metabolism. (A) Oil Red O staining of HepG2 cells treated with different FFA concentrations; scale bar = 50 μm. (B) CCK8 detects the cell viability of HepG2 cells treated with different FFA concentrations (*n* = 6, where *n* represents the number of cell samples, and all the cells in each well plate constitute one cell sample). (C) CCK8 detects the cell viability of HepG2 cells treated with different drugs (*n* = 3, where *n* represents the number of cell samples, and all the cells in each well plate constitute one cell sample). (D) Oil Red O staining of FFA‐induced HepG2 cells treated with different drugs. (E) The lipid content of FFA‐induced HepG2 cells treated with different drugs detected by OD_510_ value (*n* = 3, where *n* represents the number of cell samples, and all the cells in each well plate constitute one cell sample). (F) Relative TG content in FFA‐induced HepG2 cells treated with different drugs (*n* = 3, where *n* represents the number of cell samples, and all the cells in each well plate constitute one cell sample). The data are expressed as mean ± SD and analyzed by one‐way ANOVA with Duncan's multiple range test for comparative experiments. *P* < 0.05 was considered statistically significant.

To ascertain the optimal concentration and time for FFA (i.e. a mixture of sodium oleate and sodium palmitate at a ratio of 2:1)‐induced cellular steatosis, Oil Red O staining (Fig. 3A) and cytotoxicity assays (Fig. 3B) were performed. With reference to the relevant literature [26] and the experimental requirements, the conditions for model establishment in the subsequent experiments were determined as follows: the concentration of FFA was 1 mm and the duration of model establishment was 24 h. In the subsequent experiments, the cytotoxicity of CA, BBR, AT and BBR‐CA, respectively, on HepG2 cells was evaluated. The results demonstrated that no significant cytotoxicity was observed within the concentration range up to 60 μm (Fig. 3C). The Oil Red O staining results, as presented in Fig. 3D,E, indicated that BBR‐CA was capable of markedly reducing intracellular lipid accumulation. Quantitative analysis further revealed that the efficacy of BBR‐CA was substantially superior to that of CA and BBR. The AT group also significantly reduced the lipid droplet area in FFA‐induced HepG2 cells, and its *D*_510_ value (lipid content) was close to that of the H‐BBR‐CA group (Fig. 3E). This indicates that the positive control in this experimental system is effective, and the lipid‐lowering effect of BBR‐CA is comparable to that of clinical drugs. Additionally, BBR‐CA also led to a significant reduction in the intracellular TG levels (Fig. 3F). Collectively, the above results suggest that BBR‐CA can significantly ameliorate FFA‐induced lipid accumulation in HepG2 cells.

### BBR‐CA reduces the expression of lipid synthesis proteins in HepG2 cells

**Fig. 4:**
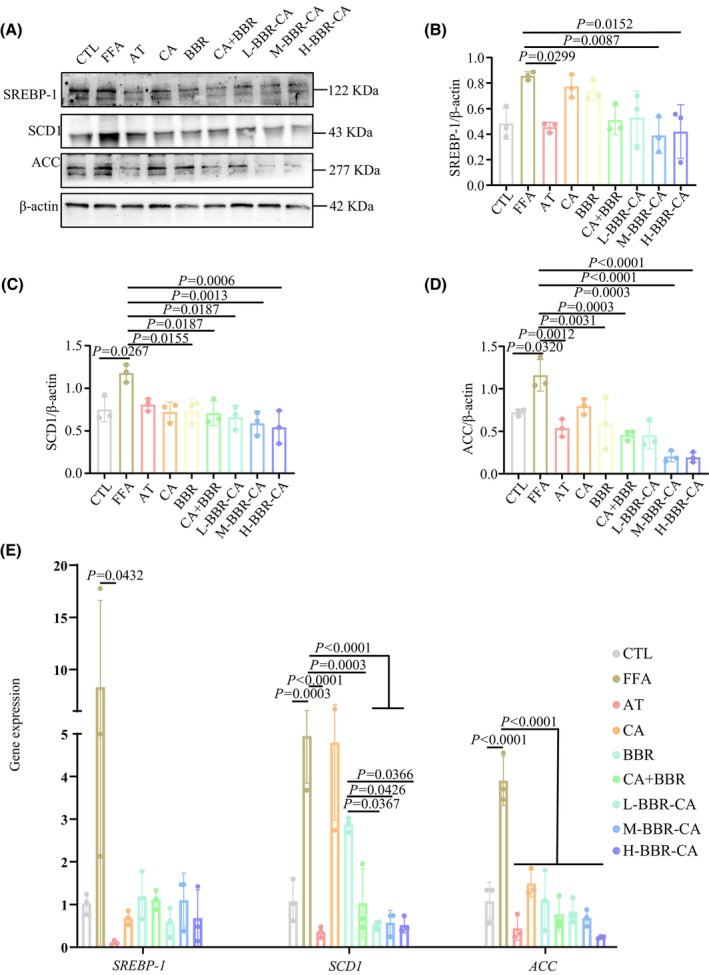
The effects of BBR‐CA on the expression of lipid synthesis‐related proteins and genes in HepG2 cells. (A) Western blotting detects the protein expression of SREBP‐1, SCD1 and ACC in HepG2 cells. (B–D) Quantification of the SREBP‐1, SCD1 and ACC bands from (A) (*n* = 3, where *n* represents the number of cell samples, and all the cells in each well plate constitute one cell sample). (E) Changes in mRNA expression of *SREBP‐1*, *SCD1* and *ACC* in HepG2 cells (*n* = 3, where *n* represents the number of cell samples, and all the cells in each well plate constitute one cell sample). The data are expressed as mean ± SD and analyzed by one‐way ANOVA with Duncan's multiple range test for comparative experiments. *P* < 0.05 was considered statistically significant.

In the cell experiments, western blot assays were utilized to determine the protein expression levels of SREBP‐1, SCD1 and ACC in cells of each experimental group. In comparison with the CTL group, a significant elevation in the protein expression levels of SREBP‐1, SCD1 and ACC was observed in the FFA group (Fig. 4). Subsequently, following drug administration in each group, this trend was notably reversed. Specifically, the combined application of CA and BBR demonstrated a more pronounced effect compared to single‐agent administration, and BBR‐CA alone also exhibited enhanced efficacy. When contrasted with the CA + BBR group, the medium and high doses of BBR‐CA manifested a more conspicuous effect with respect to reducing the levels of lipid synthesis proteins within HepG2 cells (Fig. 4A–D). Further detection of the expression levels of lipid synthesis protein genes showed results similar to those of the protein levels. Compared with the CTL group, the expression levels of *SREBP‐1*, *SCD1* and *ACC* in the FFA group were significantly increased, and BBR‐CA could significantly reduce their expression levels (Fig. 4E). Meanwhile, the AT group significantly down‐regulated the protein and gene expression of SREBP‐1, SCD1 and ACC, which was consistent with the regulatory trend of the M‐BBR‐CA group. This further supports that BBR‐CA exerts lipid‐lowering effects by inhibiting lipid synthesis‐related proteins (Fig. 4A,E). Collectively, these results imply that BBR‐CA can substantially attenuate the expression levels of lipid synthesis proteins and genes in HepG2 cells induced by FFA.

### BBR‐CA decreases the SREBP‐1 in the nucleus of HepG2 cells

**Fig. 5:**
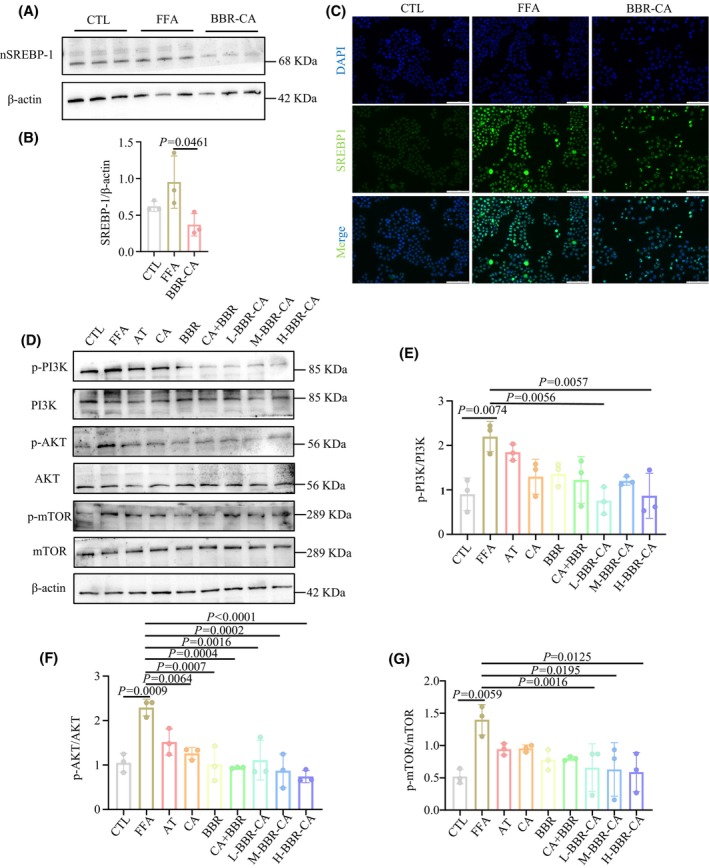
The effects of BBR‐CA on the expression of SREBP‐1 in the nucleus (nSREBP‐1) and its upstream proteins in HepG2 cells. (A, B) The effect of protein expression of BBR‐CA on nSREBP‐1 of HepG2 cells (*n* = 3, where *n* represents the number of cell samples, and all the cells in each well plate constitute one cell sample). (C) Immunofluorescence detects the localization of SREBP‐1 in HepG2 cells. (D) Western blotting detects the protein expression of p‐PI3K/PI3K, p‐AKT/AKT and p‐mTOR/mTOR in HepG2 cells. (E–G) Quantification of the p‐PI3K/PI3K, p‐AKT/AKT and p‐mTOR/mTOR bands from (D) (*n* = 3, where *n* represents the number of cell samples, and all the cells in each well plate constitute one cell sample). The data are expressed as mean ± SD and analyzed by one‐way ANOVA with Duncan's multiple range test for comparative experiments. *P* < 0.05 was considered statistically significant.

The regulation of lipid synthesis by SREBPs is a complex process and provides targets to cue hyperlipidemia. Precursor SREBPs are formed on the endoplasmic reticulum and transported to the Golgi apparatus together with SREBP cleavage‐activating protein. After being cleaved by site‐1 protease and site‐2 protease, the active N‐terminal of SREBP‐1 enter the nucleus and bind to SRE response elements to initiate the expression of lipogenic genes for SCD1 and ACC. The expression of SREBP‐1 in the nucleus was detected by western blotting and immunofluorescence methods. The results of western blotting showed that the expression level of SREBP‐1 in the nucleus of the FFA group was higher than that in the CTL group, and the expression level of SREBP‐1 decreased after the administration of BBR‐CA (Fig. 5A,B). In terms of immunofluorescence, green fluorescence was mainly distributed in the nucleus, indicating that SREBP‐1 was mainly located in the nucleus. After the administration of BBR‐CA, the fluorescence intensity in the nucleus decreased significantly (Fig. 5C). The above results suggest that BBR‐CA can affect hyperlipidemia by reducing the expression of SREBP‐1 in the nucleus.

### BBR‐CA reduces the lipid accumulation in HepG2 cells by affecting the PI3K/AKT/mTOR pathway

Studies have shown that mTOR regulates downstream phosphorylated (p)‐70S6K to promote the cleavage and activation of SREBPs from the endoplasmic reticulum to the Golgi apparatus [27, 28]. PI3K is the initiator of the PI3K/AKT/mTOR signaling pathway, and, among them, class I PI3K is an essential link for activating the mTOR signaling pathway. AKT, as the signal transduction hub and an important downstream target protein of the PI3K/AKT/mTOR signaling pathway, plays a significant role in the homeostatic regulation of cell growth, survival and glucose metabolism. The activated AKT then translocates to the cytoplasm or nucleus again and regulates downstream signaling molecules [29]. The PI3K/AKT/mTOR pathway can modulate the expression of SREBP‐1 and downstream lipid synthesis proteins [17]. Hence, we investigated the expression of the PI3K/AKT/mTOR pathway in HepG2 cells. As depicted in Fig. 5D–G, the model under the influence of FFA enhanced the expression of p‐PI3K, p‐AKT and p‐mTOR. Upon treatment with all drugs, the phosphorylation levels of PI3K, AKT and mTOR were significantly diminished, with BBR‐CA exhibiting the most pronounced effect. These results imply that BBR‐CA can attenuate the expression of the PI3K/AKT/mTOR signaling pathway, thereby repressing the expression of SREBP‐1.

**Fig. 6:**
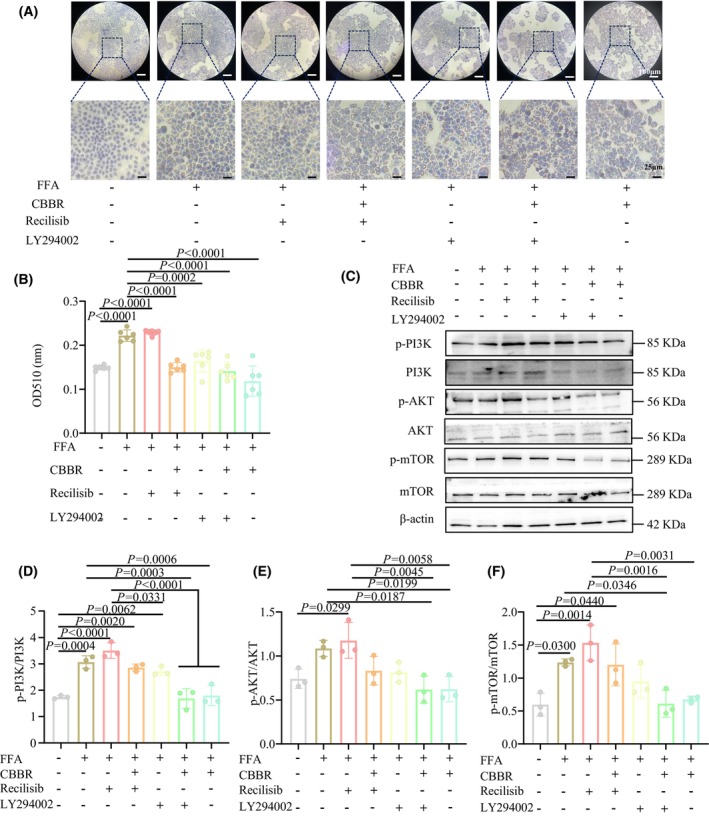
The effects of PI3K agonist and inhibitor on BBR‐CA‐reduced lipid synthesis in HepG2 cells. (A) Oil Red O staining of HepG2 cells treated with different drugs; scale bar = 50 μm. (B) The lipid content of FFA‐induced HepG2 cells treated with different drugs detected by OD_510_ value (*n* = 6, where *n* represents the number of cell samples, and all the cells in each well plate constitute one cell sample). (C) Western blot detects the protein expression of p‐PI3K/PI3K, p‐AKT/AKT and p‐mTOR/mTOR in HepG2 cells treated with different drugs. (D–F) Quantification of the p‐PI3K/PI3K, p‐AKT/AKT and p‐mTOR/mTOR bands from (C) (*n* = 3, where *n* represents the number of cell samples, and all the cells in each well plate constitute one cell sample). The data are expressed as mean ± SD and analyzed by one‐way ANOVA with Duncan's multiple range test for comparative experiments. *P* < 0.05 was considered statistically significant.

To further confirm that BBR‐CA can indeed modulate the PI3K/AKT/mTOR pathway, we treated HepG2 cells with a PI3K agonist recilisib and an inhibitor LY294002. The results showed that the intracellular lipid changed after the administration of the agonist and inhibitor. Compared with the CTL group, the number of intracellular lipids increased in the FFA group and the agonist group, and this phenomenon was reversed after the administration of BBR‐CA (Fig. 6A,B). In addition, in FFA‐induced HepG2 cells, the expression levels of p‐PI3K, p‐AKT and p‐mTOR increased significantly. After treatment with the inhibitor, the expression levels of p‐PI3K, p‐AKT and p‐mTOR in HepG2 cells decreased (Fig. 6C–F). After the administration of BBR‐CA, the PI3K activator reversed the effect of BBR‐CA, and the inhibitor of PI3K enhanced the down‐regulatory effect of BBR‐CA on protein phosphorylation (Fig. 6C–F). In conclusion, these results indicate that BBR‐CA can affect lipid synthesis by regulating the phosphorylation of proteins in the PI3K/AKT/mTOR pathway.

## Discussion

CA, one of the active constituents of cinnamon, and BBR, a major component of *Coptis chinensis*, have attracted considerable attention in recent years as a result of their abundant pharmacological activities. In previous studies, CA and BBR have been demonstrated to possess the functions of regulating metabolism and lipid metabolism [30, 31]. Particularly, BBR has drawn increasing attention regarding its application and development in metabolic diseases in recent years [32]. However, its low bioavailability has significantly limited the application of BBR [33]. In the present study, CA was used as a ligand to combine BBR with cinnamic acid and self‐assemble to form a co‐crystal (1:1), aiming to enhance the pharmacological efficacy of BBR and explore the mechanism of action of BBR‐CA in hyperlipidemia. In the mouse model, BBR‐CA remarkably inhibited the increase in body weight of mice, reversed the lipid disorder in mice and reduced the lipid accumulation in the liver, with the effect being superior to that of BBR alone (Fig. 1). Although multiple studies have demonstrated that CA has a wide range of pharmacological activities, in the preliminary studies, the effect of CA at the same concentration as BBR was not obvious. Therefore, we excluded the CA group in the animal experiment grouping, which is one of the limitations of this study. Additionally, the co‐crystallized structure has higher stability and a tighter crystal lattice, which can enhance the stability and bioavailability of the drug. Meanwhile, co‐crystallization enables rapid dissolution and drug release, shortening the onset time of the drug action and thus improving the therapeutic effect. Therefore, the better efficacy of BBR‐CA in the present study may be attributed to the advantages of co‐crystallization, the synergistic effect of the two drugs, or both, leading to an unclear mechanism in this regard. In the future, we aim to comprehensively investigate the *in vivo* and *in vitro* efficacy and mechanism of BBR‐CA from multiple perspectives, including drug concentration in blood, absorption efficiency and action targets.

Interestingly, the level of HDL‐C in the serum of mice induced by a HFD was higher compared to that of mice in the normal diet group, which is similar to some previously reported studies [30, 34]. A possible reason for this is that when mice are in a state of hyperlipidemia, there is an imbalance in the metabolism of cholesterol in the body. In response to this situation, the liver compensatorily increases the synthesis of HDL. The main function of HDL is to transport cholesterol from peripheral tissues, such as those in the arterial wall, back to the liver for metabolism, which is commonly referred to as reverse cholesterol transport. In this way, HDL can reduce the deposition of cholesterol in the body, thereby alleviating the adverse effects caused by hyperlipidemia. Because the experimental period in our study was 8 weeks, and the modeling time was relatively short, corresponding to the early stage of hyperlipidemia, this also explains why there were no significant changes in the levels of transaminases in the liver and no obvious toxic and side effects were observed in other organs. However, as the disease progresses and reaches the late stage, this compensatory mechanism may fail, and the level of HDL‐C may decrease.

SREBP‐1 is a crucial transcription factor that regulates the expression of genes related to fatty acid, TG and phospholipid synthesis [35]. During the initiation of lipid synthesis, SREBP‐1, as a homodimer, is transported from the endoplasmic reticulum to the nucleus, where it binds to the SRE sequence and stimulates the transcription of downstream target genes, such as ACC and SCD1, and promotes the synthesis of endogenous free fatty acids [36]. SCD1 is a rate‐limiting lipase that plays an essential role in catalyzing the synthesis of monounsaturated fatty acids (mainly oleate and palmitate). From a biological perspective, the overexpression of SCD1 can lead to numerous metabolic disorders, including obesity, insulin resistance, hypertension and hypertriglyceridemia [37]. *De novo* fatty acid synthesis is an important metabolic process that generates metabolic intermediates for energy storage, membrane lipid biosynthesis and signal molecule production. ACC is a key enzyme in fatty acid synthesis, which carboxylates acetyl‐CoA to form malonyl‐CoA. The role of ACC in fatty acid synthesis makes it a promising therapeutic target for various metabolic diseases such as non‐alcoholic fatty liver disease, obesity and diabetes [38]. In this study, both *in vivo* and *in vitro* experiments have shown that BBR‐CA significantly reduced the expression of SREBP‐1 as well as the downstream SCD1, and also ACC. Moreover, BBR‐CA also inhibited the expression of SREBP‐1 in the cell nucleus, thereby reducing lipid synthesis. This may be because BBR‐CA affects the transfer of SREBP‐1 from the endoplasmic reticulum to the cell nucleus to exert its function.

As described in previous studies, the PI3K/AKT/mTOR signaling pathway, incorporating the PI3K/AKT pathway and its primary downstream target mTOR, is of vital importance in the regulation of lipid metabolism. SREBP‐1 becomes activated via the PI3K/AKT pathway. Furthermore, the mammalian target of rapamycin complex 1 (i.e. mTORC1) is a key component of this pathway. When hepatocytes express constitutively active AKT, the pathway becomes activated; this leads to greater accumulation of the mature SREBP‐1 form and stimulates hepatic lipid synthesis through *de novo* fatty acid production [20, 39, 40]. A large body of research has shown that irregular regulation of the PI3K/AKT signaling pathway causes an upsurge in *de novo* lipogenesis. This, in turn, intensifies the flow of non‐esterified FFA in the liver by boosting lipolysis, eventually resulting in non‐alcoholic fatty liver disease [41, 42]. Likewise, a prior study focusing on hepatocellular carcinoma revealed that activating the AKT/mTOR pathway was able to raise SREBP‐1 expression levels, thereby restructuring hepatic lipid metabolism [43]. In subsequent experiments, BBR‐CA reduced the expression levels of phosphorylated PI3K/AKT/mTOR in FFA‐induced HepG2 cells. Moreover, in the experiments involving the administration of PI3K activators and inhibitors, it was demonstrated that BBR‐CA indeed acts on PI3K. By regulating the PI3K/AKT/mTOR signaling pathway, BBR‐CA decreases the expression of SREBP‐1, reduces *de novo* lipid synthesis and thus treats hyperlipidemia.

## Conclusions

In conclusion, BBR‐CA has been demonstrated to possess a favorable effect in treating hyperlipidemia through both *in vivo* and *in vitro* experiments and shows good potential for development. Moreover, the mechanism underlying its treatment of hyperlipidemia may be through regulating the PI3K/AKT/mTOR/SREBP‐1 pathway to inhibit *de novo* lipid synthesis.

## Conflicts of interest

The authors declare that they have no conflicts of interest.

## Author contributions

WG and YL were responsible for writing the original draft. WG, YL, LC and WY were responsible for methodology. WG, YL and WY were responsible for visualization. WG, YL, LC and ST were responsible for data curation. WG and YL were responsible for conceptualization. YY, DY and ST were responsible for reviewing and editing. ST was responsible for funding acquisition. All authors have read and approved the final version of the manuscript submitted for publication.

## Supporting information


**Fig. S1.** Hematoxylin and eosin (H&E) staining of heart, spleen, lung and kidney in mice (200×).
**Table S1.** List of primary antibodies used for western blot and immunohistochemistry.
**Table S2.** Primer sequences for qPCR.

## Data Availability

The data that support the findings of this study are available from the corresponding author upon reasonable request.
