# Supplementary material for: Berberine–cinnamic acid co‐crystal effect in ameliorating hyperlipidemia might be regulated through the PI3K/AKT/mTOR/SREBP‐1 signaling pathway
Source: FEBS Open Bio. 2025 Sep 10;16(1):145–60. doi: 10.1002/2211-5463.70115 (PMC12767761; doi:10.1002/2211-5463.70115)
Supplement: Supplementary file 1 — Fig. S1. Hematoxylin and eosin (H&E) staining of heart, spleen, lung and kidney in mice (200×). Table S1. List of primary antibodies used for western blot and immunohistochemistry. Table S2. Primer sequences for qPCR. [file FEB4-16-145-s001.docx]

**Berberine-cinnamic acid co-crystal effect in ameliorating hyperlipidemia might be regulated through the PI3K/AKT/mTOR/SREBP-1 signaling pathway**

Wenheng Gao^a^, Yunlong Li^a^, Lihua Chen^a^, Wenshuo Yang^a^, Yong He^b^, Ye Yang^a, b, c *^, Dengke Yin^a, b, c, *^, Song Tan^a*^

**Table S1.** List of primary antibodies used for western blot and IF.

| Protein | Antibody | Catalog number |
| --- | --- | --- |
| β-actin | Rabbit antibody β-actin | 380624 |
| SREBP-1 | rabbit antibody SREBP-1 | 347061 |
| p-PI3K | rabbit antibody p-PI3K | 310164 |
| AKT | rabbit antibody AKT | 342529 |
| p-AKT | rabbit antibody | 310021 |
| ACC | rabbit antibody ACC | RM3588 |
| mTOR | rabbit monoclonal antibody mTOR (1L6) | RM4316 |
| Phospho-mTOR | rabbit antibody Phospho-mTOR (S2481) (4Y3) | RM4964 |
| PI3K | rabbit antibody PI3K | BD-PT6156 |
| SCD | rabbit antibody SCD | 28678-1-AP |
| HRP-IgG | horseradish peroxidase (HRP)-conjugated goat antirabbit IgG | 550010 |

**Table S2.** Primer sequences for qPCR

| Genes | Species | Sequences (5’→3’) | |
| --- | --- | --- | --- |
| *β-actin* | Mouse | Forward primer | GTGACGTTGACATCCGTAAAGA |
|  |  | Reverse primer | GTAACAGTCCGCCTAGAAGCAC |
| *SREBP1* | Mouse | Forward primer | GCCGGCGCCATGGACGAGCTGG |
|  |  | Reverse primer | CAGGAAGGCTTCCAGAGAGGAG |
| *SCD1* | Mouse | Forward primer | TTCTTGCGATACACTCTGGTGC |
|  |  | Reverse primer | CGGGATTGAATGTTCTTGTCGT |
| *ACC1* | Mouse | Forward primer | GATGAACCATCTCCGTTGGC |
|  |  | Reverse primer | GACCCAATTATGAATCGGGAGTG |
| *GAPDH* | Homo sapiens | Forward primer | ACAGTGACTTCCCTGGCCTAT |
|  |  | Reverse primer | GCATGGACGGGTACATCTTCAA |
| *SREBP1* | Homo sapiens | Forward primer | ACAGTGACTTCCCTGGCCTAT |
|  |  | Reverse primer | GCATGGACGGGTACATCTTCAA |
| *SCD1* | Homo sapiens | Forward primer | TCTAGCTCCTATACCACCACCA |
|  |  | Reverse primer | TCGTCTCCAACTTATCTCCTCC |
| *ACC1* | Homo sapiens | Forward primer | ATGTCTGGCTTGCACCTAGTA |
|  |  | Reverse primer | CCCCAAAGCGAGTAACAAATTCT |


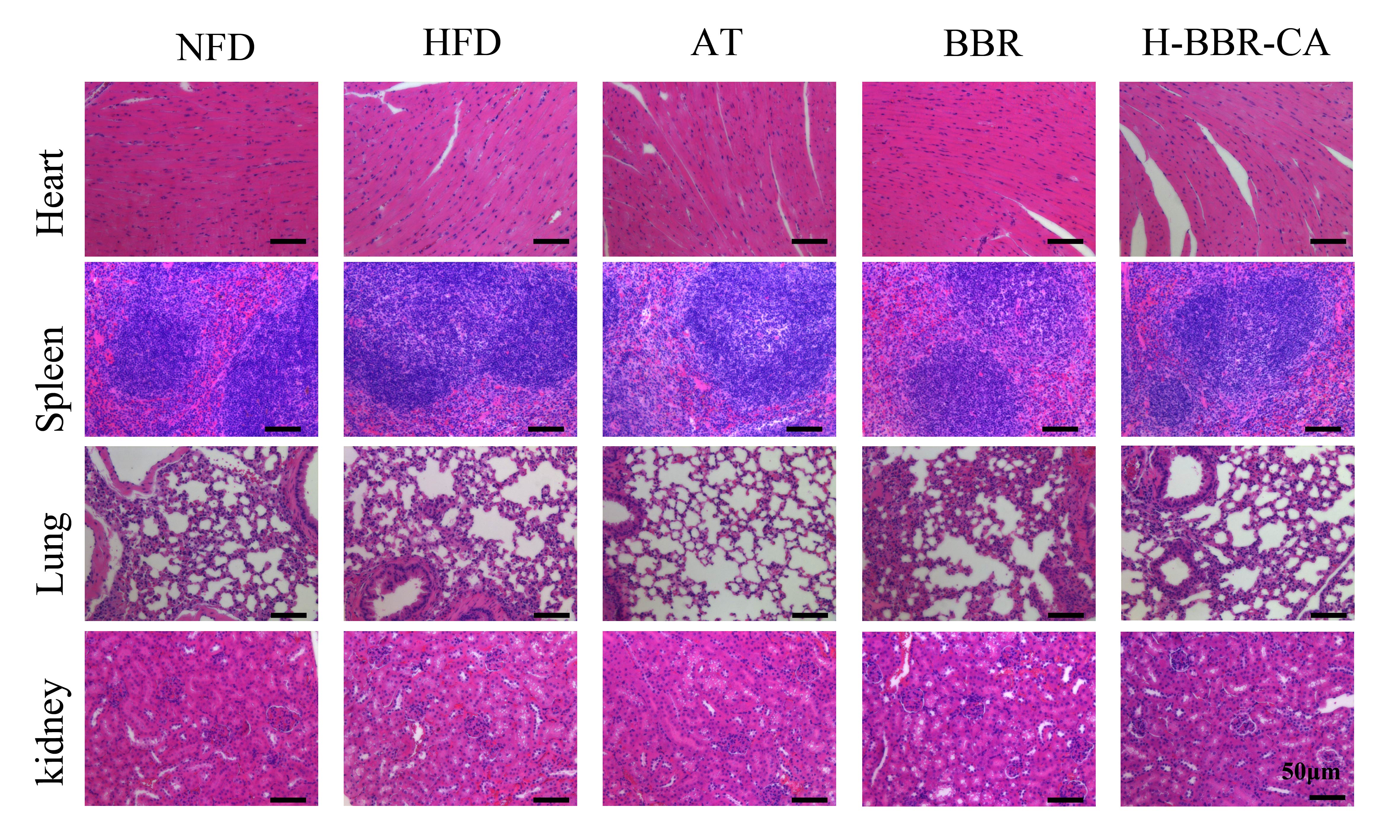


**Figure S1** Hematoxylin-eosin (H&E) staining of heart, spleen, lung and kidney in mice (200 ×)
